# Non-invasive continuous monitoring of pro-oxidant effects of engineered nanoparticles on aquatic microorganisms

**DOI:** 10.1186/s12951-017-0253-x

**Published:** 2017-03-07

**Authors:** Christian Santschi, Nadia Von Moos, Volodymyr B. Koman, Vera I. Slaveykova, Paul Bowen, Olivier J. F. Martin

**Affiliations:** 1 Nanophotonics and Metrology Laboratory (NAM), École Polytechnique Fédéral de Lausanne, EPFL/IST/IMT/NAM, Station 11, 1015 Lausanne, Switzerland; 2 Environmental Biogeochemistry and Ecotoxicology, Department F.-A. Forel for Environmental and Aquatic Sciences, Earth and Environmental Sciences, Faculty of Sciences, University of Geneva, 66, Bvd Carl-Vogt, 1211 Geneva, Switzerland; 3 Powder Technology Laboratory (LTP), École Polytechnique Fédéral de Lausanne, 1015 Lausanne, Switzerland

**Keywords:** Ecotoxicity, Nanomaterials, Reactive oxygen species, Oxidative stress, Hydrogen peroxide, Optical biosensor, Multiscattering, Absorption spectroscopy

## Abstract

Engineered nanomaterials (ENMs) are key drivers for the development of highly sophisticated new technologies. As all new attainments, the rapidly increasing used of ENMs raise concerns about their safety for the environment and humans. There is growing evidence showing that if engineered nanomaterials are released into the environment, there is a possibility that they could cause harm to aquatic microorganisms. Among the divers effects triggering their toxicity the ability of ENMs to generate reactive oxygen species (ROS) capable of oxidizing biomolecules is currently considered a central mechanism of toxicity. Therefore, development of sensitive tools for quantification of the ROS generation and oxidative stress are highly sought. After briefly introducing ENMs-induced ROS generation and oxidative stress in the aquatic microorganisms (AMOs), this overview paper focuses on a new optical biosensor allowing sensitive and dynamic measurements of H_2_O_2_ in real-time using multiscattering enhanced absorption spectroscopy. Its principle is based on sensitive absorption measurements of the heme protein cytochrome *c* whose absorption spectrum alters with the oxidation state of constituent ferrous Fe^II^ and ferric Fe^III^. For biological applications cytochrome *c* was embedded in porous random media resulting in an extended optical path length through multiple scattering of light, which lowers the limit of detection to a few nM of H_2_O_2_. The sensor was also integrated in a microfluidic system containing micro-valves and sieves enabling more complex experimental conditions. To demonstrate its performance, abiotic absorption measurements of low concentrations of dye molecules and 10 nm gold particles were carried out achieving limits of detection in the low nM range. Other biologically relevant reactive oxygen species can be measured at sub-μM concentrations, which was shown for glucose and lactate through enzymatic reactions producing H_2_O_2_. In ecotoxicological investigations H_2_O_2_ excreted by aquatic microorganisms exposed to various stressors were measured. Pro-oxidant effects of nano-TiO_2_ and nano-CuO towards green alga *Chlamydomonas reinhardtii* were explored in various exposure media and under different light illuminations. Dynamics of Cd^2+^ induced effects on photosynthetic activity, sensitisation and recovery of cells of *C. reinhardtii* was also studied.

## Background

The material revolution engendered by nanotechnological advances in the last decades has not only enabled the development of highly sophisticated fine-tuned materials for new applications but also confronted established risk assessment and regulatory affairs with new challenges: the possible (eco-)toxicological implications of the expected increment of engineered nanomaterials (ENMs) discharged into environmental compartments [1].

**Fig. 1:**
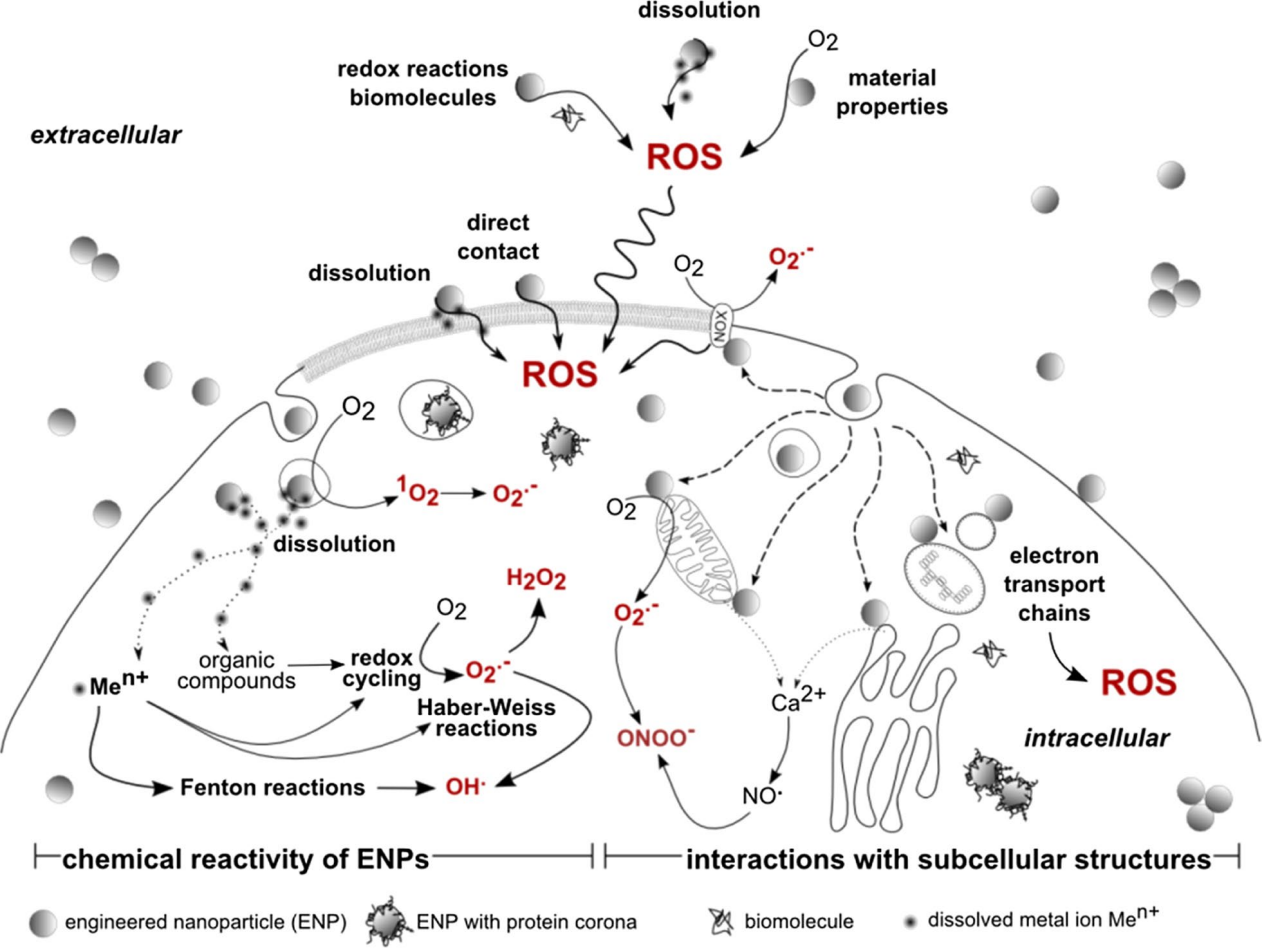
Mechanisms of ROS generation by engineered nanomaterials via intracellular chemical reactivity (*left hand side*) or via physical interactions with subcellular compartments (*right hand side*). ENPs generate ROS by direct and indirect chemical reactions. Direct reactions involve the photoexcitation of O_2_, which yields singlet oxygen (^1^O_2_) and superoxide (O_2_^·−^). Indirect chemical reactions involve reactions between leached ENP constituents (e.g. metal ions, organic compounds) that engage in redox cycling that yields superoxide (O_2_^·−^) and hydrogen peroxide (H_2_O_2_) or in hydroxyl radical (OH^·^) producing Fenton and Haber–Weiss reactions. ROS yielding interactions encompass the interference with electron transfer chains in chloroplasts, peroxisomes, mitochondria and the endoplasmatic reticulum. Furthermore, interactions of ENPs and mitochondria or the endoplasmatic reticulum can also cause a loss of organelle membrane integrity that triggers the release of Ca^2+^ ions from interior stores, which may activate ROS generating Ca^2+^/calmodulin-dependent enzymes, i.e. certain nitrogen monoxide synthase isoforms that produce NO^·^. Interactions with NADPH oxidase (NOX) complexes in the cell membrane yield O_2_^·−^ [29]. Illustration adapted from Unfried, Albrecht [29], not to proportion. Reprinted with permission from (*Nanotoxicology* 2014; 8: 605–630). Copyright (2014)

Natural water bodies, one environmental sink of discharged ENMs, are estimated to receive 0.4–7% of the total global mass flow of ENMs [2]. Once in the aquatic systems ENMs interact with different biotic and abiotic components and potentially harm different organisms [3]. There is currently an agreement [4] that three major phenomena drive the detrimental effects of the ENMs to aquatic organisms: (i) their dissolution, (ii) their organism-dependent cellular uptake and (iii) the induction of oxidative stress and consequent cellular damages. The ability of ENMs to generate reactive oxygen species (ROS) capable of oxidizing biomolecules is currently considered a central (but by no means sole) mechanism of toxicity, potentially leading to oxidative stress and damage (Fig. 1) [5–12].

It is postulated that increased levels of ROS and oxidative damage will occur in exposed organisms (despite the presence of basal or enhanced antioxidant defence systems of repair and replacement), which may be linked to some aspect of impaired biological functions at cellular or higher levels of organization [13]. Thus, from the nanoecotoxicological perspective seeking the elucidation of environmental hazards of ENMs, it follows that an in-depth understanding of their toxic mode of action, that is, of normal and ENM-stimulated ROS production as well as antioxidant levels in aquatic organisms is required. This will allow to quantitatively link the presence of ENMs with pro-oxidant processes and to estimate the expected degree by which ENM-stimulated oxidative damage may potentially affect overall health of organism.

Hence, there has been a keen interest in the detection and quantification of ROS in aqueous and biological systems, which is a technically tricky task due to their very low concentration in the pico- to micromolar range and their extremely short-lived nature with half times ranging from nanoseconds to hours [14]. Most conventional ROS sensing methods rely on exogenous probes or resulting endogenous reaction products and molecular biomarkers reflecting oxidative damage and antioxidant status [13, 15–17]; they suffer one major technical drawback—the invasive nature of the detection method itself [18].

The present article provides an overview of the main findings of the project “Non-invasive continuous monitoring of the interaction between nanoparticles and aquatic microorganisms” within in the framework of the Swiss National Research Program 64 on the Opportunities and Risk of Nanomaterials. The review begins with a brief introduction in the ENMs-induced ROS generation and oxidative stress in the aquatic microorganisms (AMOs) as well as short presentation of the existing detection techniques. The newly developed method for non-invasive quantification of extracellular H_2_O_2_ in real-time and monitoring with an unprecedented limit of detection is described, while its capabilities are illustrated by exploring the pro-oxidants effects of the ENMs to AMOs [18].

## ENMs and oxidative stress in aquatic microorganisms

Investigations performed in the mid-90’s led to the conclusion that nanoparticles have the ability to stimulate the generation of reactive oxygen (ROS) and nitrogen species (RNS) at or near the cell surface and to induce oxidative stress [10, 12, 19]. The oxidative stress hypothesis was successfully expanded into nanotoxicology and recognised as a major mechanism for nanoparticle induced effects [23]. Therefore, the impacts of ENMs on the pro-oxidant/antioxidant equilibrium can provide relevant information on their ecotoxicical importance [5].

**Fig. 2:**
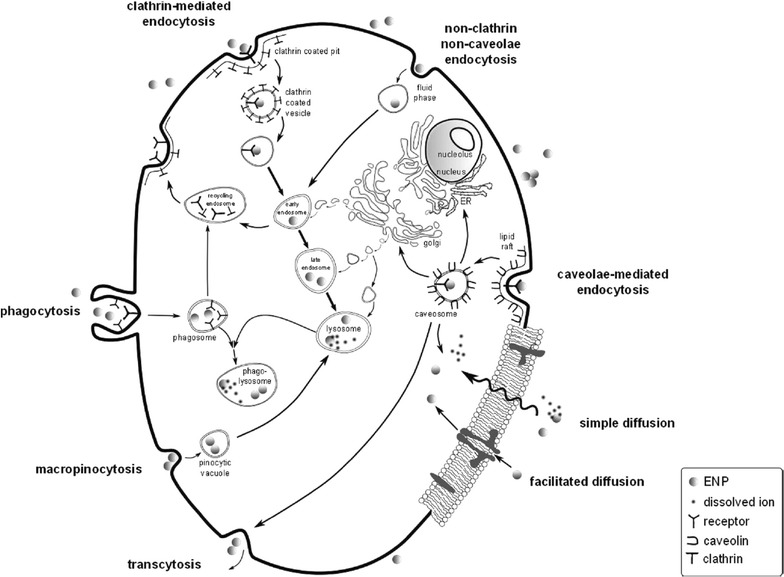
Active and passive cellular uptake pathways for ENMs in eukarotic cells. Passive uptake occurs via diffusion and facilitated diffusion via transport proteins, i.e. gated channel proteins and carrier proteins. Active uptake pathways involve transmembrane carrier proteins and endocytic pathways including receptor-mediated phagocytosis, clathrin-mediated endocytosis (120 nm, via clathrin-coated pits) and caveolae-mediated endocytosis (60 nm, via lipid rafts), non-specific endocytosis by macropinocytosis and non-clathrin, non-caveolae endocytosis (90 nm, fluid phase). All pathways except caveolae-mediated endocytosis and diffusion merge with the lysosomal degradation system comprising numerous vesicle maturation steps within the cell. A lysosome typically ranges from 200 to 500 nm in diameter. Phagocytosis is mediated by specific membrane-receptors that are activated upon contact with a ligand to produce phagosomes (>250 nm). During their maturation process, phagosomes transform into late phagosomes, which fuse with lysosomes to form phagolysosomes. During macropinocytosis, internalisation occurs via an unspecific invagination resulting in pinocytic vesicles (<150 nm), which eventually merge with lysosomes. Clathrin-mediated endocytosis and non-clathrin, non-caveolae-mediated endocytosis produces caveosomes that either transfer their contents into the Golgi apparatus, endoplasmatic reticulum (ER) or into the cytosol or may also undergo transcytosis. Reprinted with permission from (Environmental Science-Nano 2014; 1: 214–232). Copyright (2014) Royal Society of Chemistry

The toxicity of metal and metal oxide ENMs to organisms can be classified in direct and indirect effects [20, 21]. Direct toxic effects are principally controlled by their chemical composition and surface reactivity. Indirect effects are mainly governed by physical restraints, the release of toxic ions or the production of ROS. The latter is thought to result in elevated cellular response classified as defence, pro-inflammatory effects and cytotoxicity [22]. Toxicological effects of ENMs may include (i) inflammation related to generation of ROS and oxidative stress, depletion of glutathione and accumulation of oxidised glutathione in response to ROS generation, (ii) DNA and membrane damage, protein denaturation and immune reactivity, (iii) reduction or loss in photosynthetic activity in algae and plants. Direct toxic effects require, as a prerequisite, contact and adsorption of the ENMs with the AMOs [3, 23]. Once the ENMs are adsorbed, they may penetrate through the biological membrane and, therefore, be internalised (Fig. 2). Uptake mechanisms and different pathways leading to internalisation are discussed elsewhere [3, 4, 24]. It is important to note that ENMs can be internalised without necessarily inducing cytotoxicity, meaning that ENMs are not toxic per se [25]. However, ENMs are prone to adsorption of ambient pollutants, which can be transferred into the cells by ENMs acting as carriers (Trojan Horse effect). ENMs can trigger ROS formation extra- and intracellularly by direct and indirect chemical reactions [12] (Fig. 1). The mechanisms underlying the generation of the ROS in AMOs could involve (i) the release of metal ions from ENMs, (ii) the catalytic activity of ENMs and (iii) the redox properties at the particle surface. The pro-oxidant potential of ENMs strongly dependent of their chemical and physical properties, notably chemical composition and purity, particle size, shape and the resulting relative large reactive surface area and surface chemistry [7, 14]. For metal- containing ENMs, dissolution processes leading to ion release play a major role in terms of ecotoxicity. Many transition metal ions, such as Fe^3+^, Cu^2+^, Cr^3+^ are redox active and some of them, e.g. Fe and Cu can catalyse Fenton reactions yielding biologically highly reactive hydroxyl radicals OH^·^. The Haber–Weiss reactions in the presence of super oxide ions O_2_^−^ can also reduce redox-active metal ions which further couple to the Fenton reactions. Hence, valence state and bioavailability of redox-active ions are strongly related to the generation of ROS. Numerous inorganic ENMs, such as Ag, Pt, TiO_2_, CeO_2_, ZnO, CuO, SiO_2_ and different quantum dots were shown to generate ROS and induce oxidative stress in different organisms [5, 10, 12, 26–30]. Selected examples concerning ENM-induced oxidative stress or damage in microalgae, representative for aquatic phytoplankton are given in Table 1.

**Table 1:** Selected examples of ENM-induced oxidative stress or damage in microalgae

ENM	Algae	Media	Mechanism	Reference
TiO_2_	*C. reinhardtii*	SE	Generation of ROS by photocatalysis	[31]
TiO_2_ and UV light	*C. reinhardtii*	Lake water and MOPS buffer		[32]
TiO_2_	*Chlorella* sp.	OECD	Generation of intracellular ROS by HA	[33]
CdTe/CdS	*C. reinhardtii*	MES, MOPS, HEPES	Oxdative stress	[34]
Al_2_O_3_, SiO_2_, ZnO and TiO_2_	*Chlorella* sp.	SE	ROS may not be the dominant mechanism for algal growth inhibition	[35]
Ag	*C. vulgaris, Dunaliella tertiolecta*	Growth medium BG-11	ROS induced lipid peroxidation and a decrease of cell viability	[36]
Pt	*C. reinhardtii* *P. subcapitata*	ISO 8692 medium and 4-fold diluted tris-acetate-phosphate medium	Substantial oxidative stress and negligible membrane damage; significant growth inhibition	[30]
Coated and uncoated CuO	*C. reinhardtii*	High salt medium	ROS formation may be the primary toxicity mechanism	[37]
CeO_2_	*P. subcapitata*	Standard US EPA	The oxidative activity is mediated by OH and initiation of lipid peroxidation	[38]
Core–shell CuO	*C. reinhardtii*	High salt growth medium	ROS are responsible for chlorophyll deterioration, significant decrease of PSII primary photochemistry	[39]
CuO	*C. reinhardtii*	Various media, lake water	Oxidative stress and damage of membrane integrity	[40]
CuO and light	*C. reinhardtii*	Synthetic fresh water	Chlorophyll bleaching, oxidative stress and membrane damage; CuO and UV-light has synergistic effect	[41]
TiO_2_, CdTe and QDs	*C. reinhardtii*	CM growth medium	Lipid peroxidation induced by oxidative stress, QDs and TiO_2_ exhibit different mechanisms	[42]

Photoactive ENMs including fullerenes and semiconducting metal oxides, such as TiO_2_, CuO, CeO_2_, ZnO and Al_2_O_3_, can generate ROS when illuminated [43, 44]. It has been demonstrated that these ENMs, the most prominent being TiO_2_, can activate molecular oxygen radicals, ^1^O_2_ and O_2_^−^, which belong, together with OH^·^, to the biologically most potent ROS. It is well known that those photoactive particles are primarily active at wavelength in the UV regime (<390 nm) but it has also been demonstrated in several studies that TiO_2_ is capable to induce oxidative stress in the absence of light.

Overall, environmental contaminants, including ENMs, have the capability to induce generation of ROS in AMOs and, consequently, to alter the cellular redox homeostasis leading to oxidative stress. Oxidative stress occurs as a result of (i) increase in oxidant generation, (ii) reduction of antioxidant protection and (iii) failure to repair oxidative damage [45].

## Towards development of the novel tool for non-invasive monitoring of the pro-oxidant effects of engineered nanomaterials

**Fig. 3:**
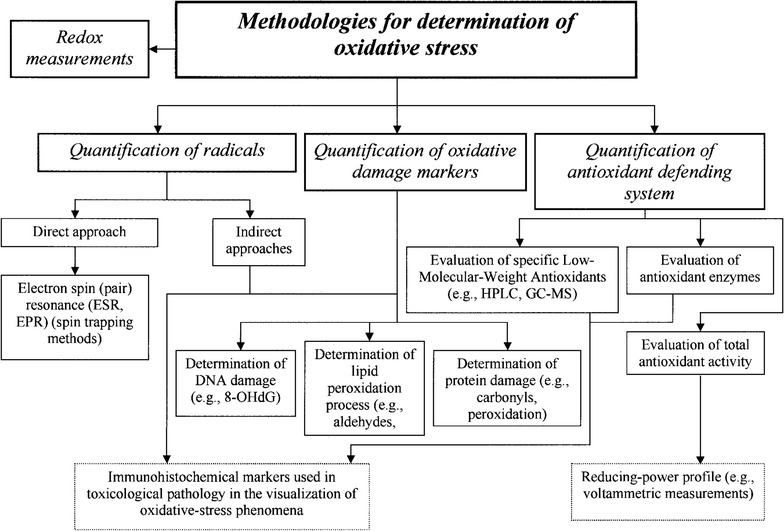
Classification of methods used to determine and quantify oxidative stress. Reprinted with permission from (Toxicologic Pathology 2002; 30: 620–650). Copyright (2002) SAGE Publications

Various approaches are available to determine oxidative stress [46]: (i) Quantification of radicals, including O_2_^−^, OH^·^ and H_2_O_2_, (ii) quantification of oxidative damage markers and (iii) quantification of antioxidants. A schematic illustration of the main approaches is displayed in Fig. 3. Superoxide O_2_^−^, represents one of the aboriginal forms of aerobic ROS. It is very reactive and short-living and can be converted to H_2_O_2_ through the reaction with SOD. H_2_O_2_ is one of the major and most stable ROS produced intracellularly by physiological and pathological processes and can cause oxidative damage. Its stability allows it to diffuse through the cell wall and can therefore be extracellularly detected [47]. Oxidative damage markers such as lipids, DNAs and proteins can be examined for alterations to quantify the extent of oxidative damage due to oxidative stress. Furthermore, several enzymes, such as SOD, CAT and GR, belonging to the antioxidative defence system, can be measured in order to quantify oxidative stress. Recent progress in fluorescent, luminescent and colorimetric ROS and RNS probes was comprehensively reviewed [48].

The above-mentioned oxidative stress “indicators” can provide a useful picture on the cell-ENM interactions. However, they are endpoint-based and qualitative, thus unable to provide quantitative information about the rate and amount of generated ROS. In addition they are often very laborious and fail to provide dynamic and continuous information on specific physiological phenomena happening at the exposed living cells.

Hereinafter a new, very sensitive detection scheme for continuous measurement of extracellular H_2_O_2_ based on multiscattering enhanced absorption spectroscopy is present. Its high sensitivity allows non-invasive and real time measurements of H_2_O_2_ related to aerobic cell activity, including oxidative stress. Stress-induced H_2_O_2_ can rapidly diffuse across plasma membranes [49, 50], is relatively long-lived (half-life 4–20 h, <1 s in living tissues) and, therefore, extracellular H_2_O_2_ could serve as an indicator of pro-oxidant processes [51–54]. A non-exhaustive list of H_2_O_2_ detection methods can be found in Table 2.

**Table 2:** Selection of H_2_O_2_ detection methods [14]

Technique/probe	Observable	LOD	Application notes	Reference
Direct detection	Absorbance of H_2_O_2_	mM		[55, 56]
Cyt *c*	Absorbance	nM	Optimal reaction in low ionic strength solutions	[57, 58]
Xylenolorange + Fe^3+^	Absorbance of complex	μM	Carried out in acidic acids	[59]
Xylenolorange + Ti^4+^	Absorbance of complex	μM	Carried out in acidic acids	[59]
Luminol	Chemi-luminescence	nM	Interference with Mn^2+^ and Fe^3+^	[60–62]
2,7-Dichlorodihydrofluorescein (DCFH)	Fluorescence of product	pM-nM	Can be oxidised by other ROS	[16, 63]
*p*-Hydroxyphenylacetic acid	Fluorescence of product	nM	Optimal reaction at pH > 8.5	[64, 65]

Fluorescent and chemi-luminescent methods exhibit low LODs in the nM range. However, a major drawback of those methods is their incompatibility with bioorganisms and they are therefore endpoint detection schemes.

## Multiscattering enhanced absorption spectroscopy (MEAS)

Thanks to its versatility, absorption spectroscopy has become a popular method with a broad range of applications. Adsorption spectroscopy provides a fast, simple and inexpensive method for the detection of a wide variety of targets [66]. Absorption spectroscopy can be applied in wide spectral span ranging from X-ray [67] to infrared light [68] and provides a beneficial tool for investigating biomolecules [69, 70]. In conventional absorption spectroscopy configurations the spectral light intensity, passed through the sample under test, is measured and normalised with respect to the intensity of the incident light. Knowing the optical path length (OPL) *l* through the sample and the absorption coefficient α of the analyte of interest, its concentration can be determined using Beer-Lambert’s law (1) [71].

1 $$\frac{I}{{I_{0} }} = e^{ - \alpha Cl}$$

I_0_ and I represent the light intensity before and after travelling through the sample, respectively. Long OPLs requires large amounts of analytes which are often costly, especially for biosamples.

**Fig. 4:**
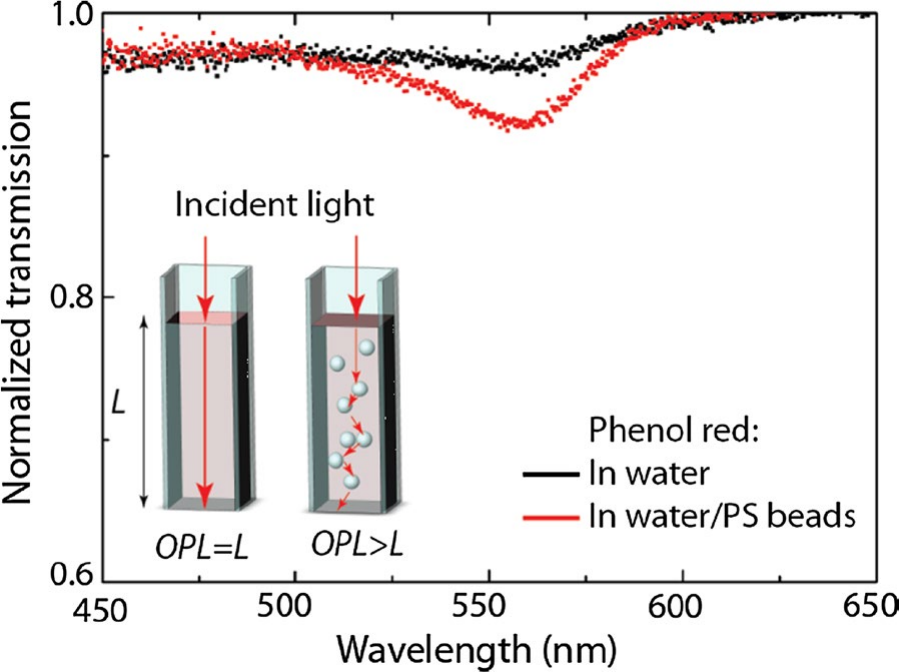
The presence of scatterers (500 nm polystyrene beads) in the MEAS configuration enhances the OPL and, consequently, lowers the LOD. Principle and transmission measurements of the absorption of phenol red in conventional and MEAS configurations. Reprinted with permission from (*Analytical Chemistry* 2015; 87: 1536–1543). Copyright (2015) American Chemical Society

Significant efforts have been put in the development of various techniques aiming to improve the sensitivity of absorption spectroscopy [72–74]. A simple and versatile technique, was presented by Koman et al. [75]. In order to extend the OPL and, thus, the sensitivity, advantages were taken from disordered media where the OPL is increased via multiple scattering since spatial variations of the refractive index prevent the light to follow the shortest trajectory. In a configuration containing suspended polystyrene (PS) beads, as schematically shown in Fig. 4, the limit of detection (LOD) was improved substantially [75].

**Fig. 5:**
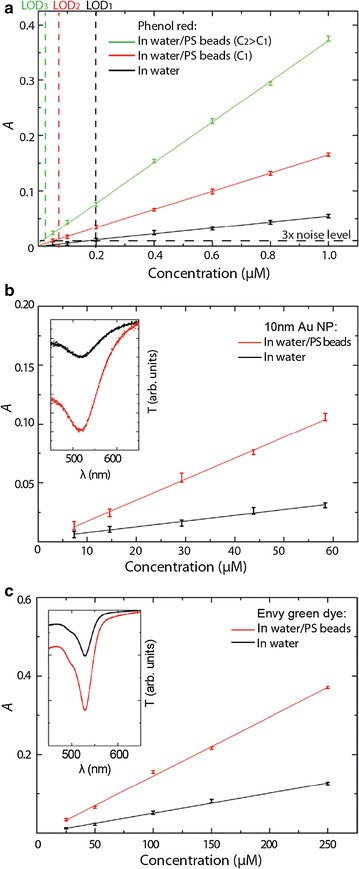
Absorption enhancement for **a** phenol *red*, **b** 10 nm Au NPs and **c** envy *green* for different concentrations C of 500 nm PS scatterers: C_1_ = 0.6 nM and C_2_ = 3 nM. The insets in **b** and **c** show the normalised transmission spectrum T and the LOD is defined as 3 times the noise level. The *error bars* correspond to the standard deviation over five independent measurements. Reprinted with permission from (*Analytical Chemistry* 2015; 87: 1536–1543). Copyright (2015) American Chemical Society

In order to demonstrate its performance MEAS was carried out on low concentrations of phenol red, envy green and 10 nm gold nanoparticles (AuNp). The absorbance A of standard and multiscattering experiments are displayed in Fig. 5 [75]. Using this approach, sensitivity and LOD of commercially available bioassays can be improved. This has been shown for OxiSelect, an assay for H_2_O_2_ detection [75].

2 $$A = - log\left( {\frac{I}{{I_{0} }}} \right) = Cl$$

According to Eq. (3) the sensitivity S for a certain analyte concentration becomes maximal. Hence, the OPL can be adjusted by selecting an adequate scatterer concentration and thereby optimised with respect to a specific application.

3 $$S = \left| {\frac{\partial }{\partial C}\frac{\Delta I}{{I_{0} }}} \right| = \alpha le^{ - \alpha Cl}$$

**Fig. 6:**
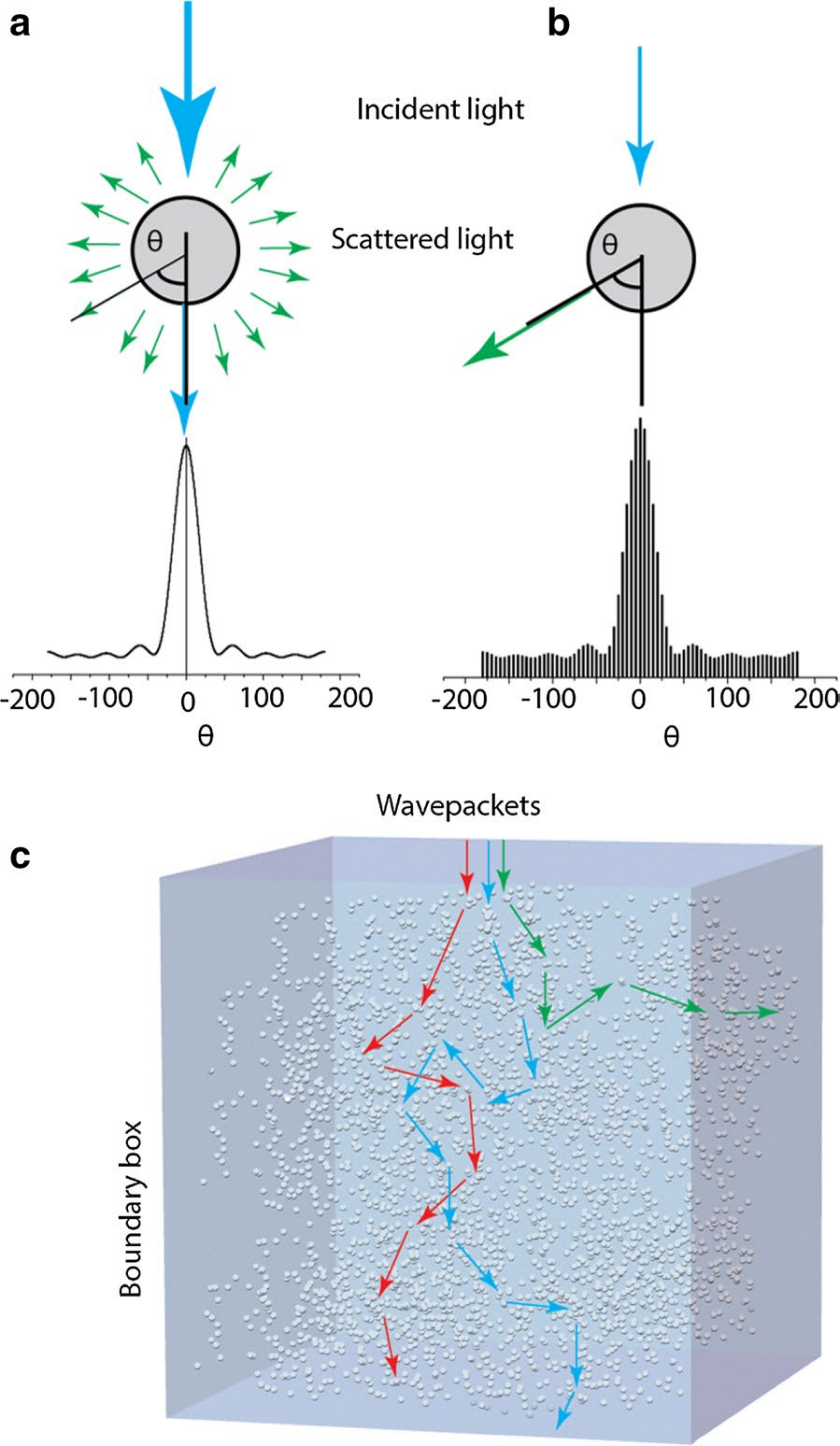
Schematic illustration of the numerical approach. **a** Intensity distribution of light scattered at a spherical object. **b** Intensity distribution of large amounts of wavepackets scattered at a spherical object. **c** Wavepackets travelling through a random media. Reprinted with permission from (*Analytical Chemistry* 2015; 87: 1536–1543). Copyright (2015) American Chemical Society

For a better understanding of the multiscattering phenomenon a probabilistic Monte Carlo approach was implemented (Fig. 6). Wavepackets are launched into the system containing randomly distributed PS beads. The random scattering angles were determined using Henyey-Greensteins approximation [76] which describes the scattering cross-section σ for an individual scatterer using Mie theory [77, 78]. The attenuation of each wavepacket was computed following Beer-Lambert’s law (1) and, finally, the residues of the individual wavepackets leaving the system were summed together. In order to achieve an appropriate accuracy the random trajectories of 10^8^ wavepackets were calculated. The simulations showed excellent agreement with experimental results and allow prediction of OPLs for different concentrations, refractive indexes and sizes of the scatterers. Due to bead–bead interactions the proposed numerical approach is not accurate for high filling factors F [79] nevertheless, for F < 10% good numerical/experimental agreements were found [75].

## Sensitive real-time detection of H_2_O_2_

**Fig. 7:**
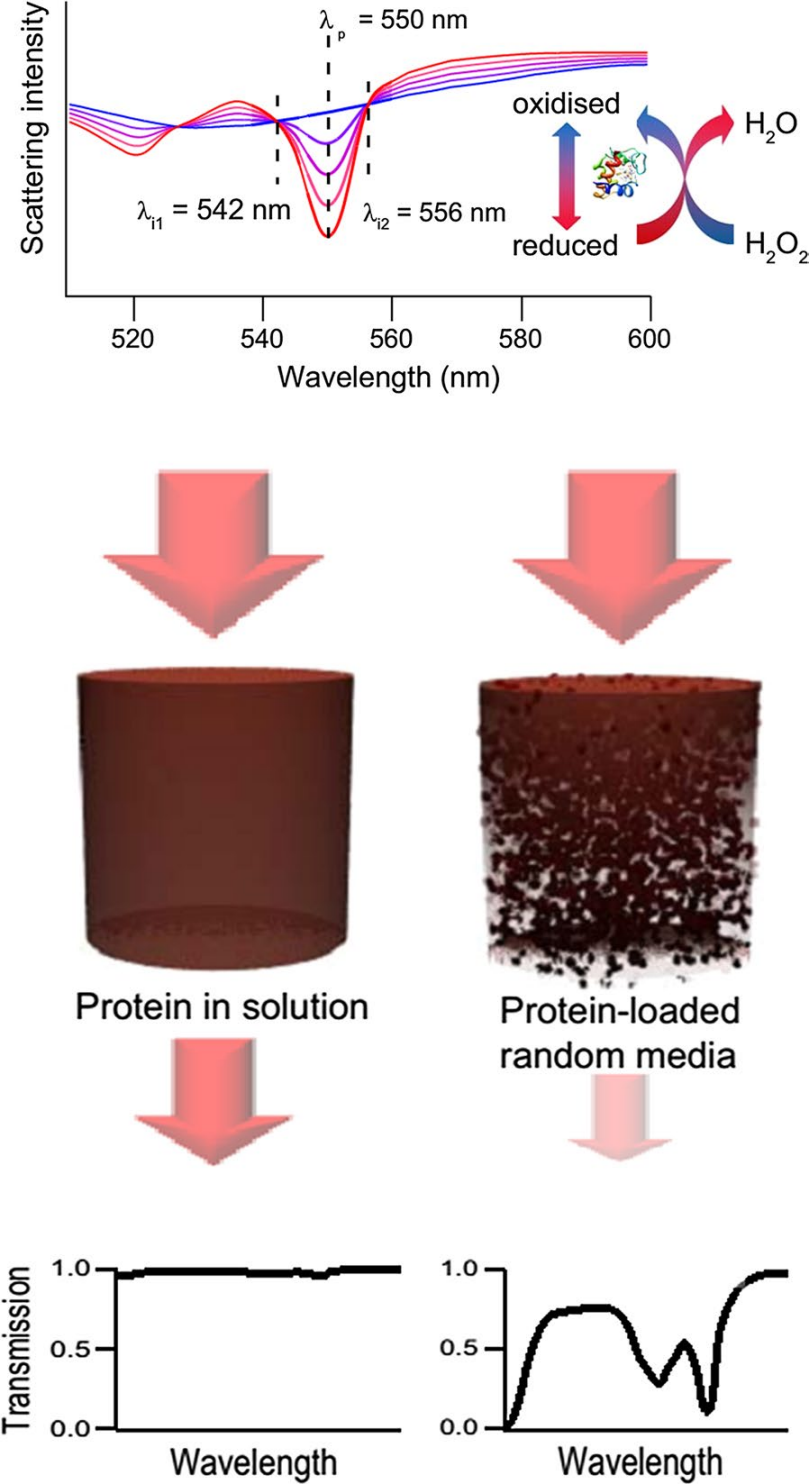
Spectrum of cyt *c* in its oxidised and reduced state. The intermediate states reflect an average value of oxidised and reduced cyt *c*. Absorption measurements in transmission configuration without and with multiscattering enhancement. Reprinted with permission from (*Scientific Reports* 2013; 3: 3447). Copyright (2013) Nature Publishing Group

MEAS was employed to improve the sensitivity for the detection of H_2_O_2_ in aqueous solutions. The detection principle is based on sensitive adsorption measurements of the heme protein cytochrome *c* (cyt *c*) [18], since the absorption spectrum of cyt *c* depends on the oxidation state of its heme group [80]. The catalytic redox behaviour of cyt *c* reduces H_2_O_2_ into water whereas the ferrous Fe^II^ heme group is oxidised into the ferric Fe^III^ heme group providing information on the H_2_O_2_ concentration in its environment. Cyt *c* exhibits three oxidation state-dependent absorption peaks in the visible range, namely, at λ = 530 nm in the oxidised and λ = 520 and λ = 550 nm in the reduced state. The absorption at λ = 542 nm and λ = 556 nm provide adequate reference signals since at those wavelengths the absorption is independent of the oxidation state (Fig. 7). The sensing molecules, cyt *c*, were embedded in a porous matrix consisting of either aggregated PS beads or a filter membrane. The aggregates were prepared as follows: PS beads were suspended in an aqueous solution of cyt *c* prior to addition of glutaraldehyde to crosslink cyt *c* resulting in cyt *c*/PS beads aggregates [18]. Transmission measurements were performed using an inverted microscope and the temporal evolution of a normalised average oxidation state coefficient φ ranging from 0 to 1 for completely oxidised and reduced cyt *c*, respectively, was determined. Calibration experiments carried out for this configuration with known concentrations of H_2_O_2_ revealed a LOD below 100 pM which enables continuous measurements of the dynamics of ROS produced by bioorganisms when undergoing stress situations [18].

Since H_2_O_2_ is the reaction product of many enzymatic reactions [Eq. (4)] [81], its real-time detection combined with those reactions enables the detection of further metabolites such as glucose and lactate.

4 $${\text{analyte }} + {\text{ enzyme }} \to {\text{ H}}_{ 2} {\text{O}}_{ 2} + {\text{ X}}$$

Koman et al. presented a detection scheme for sensitive and real-time detection of those metabolites [40]. Taking advantage of the above presented multiscattering approach they were detected with sub-micromolar LODs. Moreover, this enzymatic approach allows real-time measurements of multiple analytes in parallel which offers the possibility to follow the evolution of several metabolites. This feasibility has been demonstrated using the example of parallel detection of glucose and H_2_O_2_.

## Portable setup and microfluidic chip

**Fig. 8:**
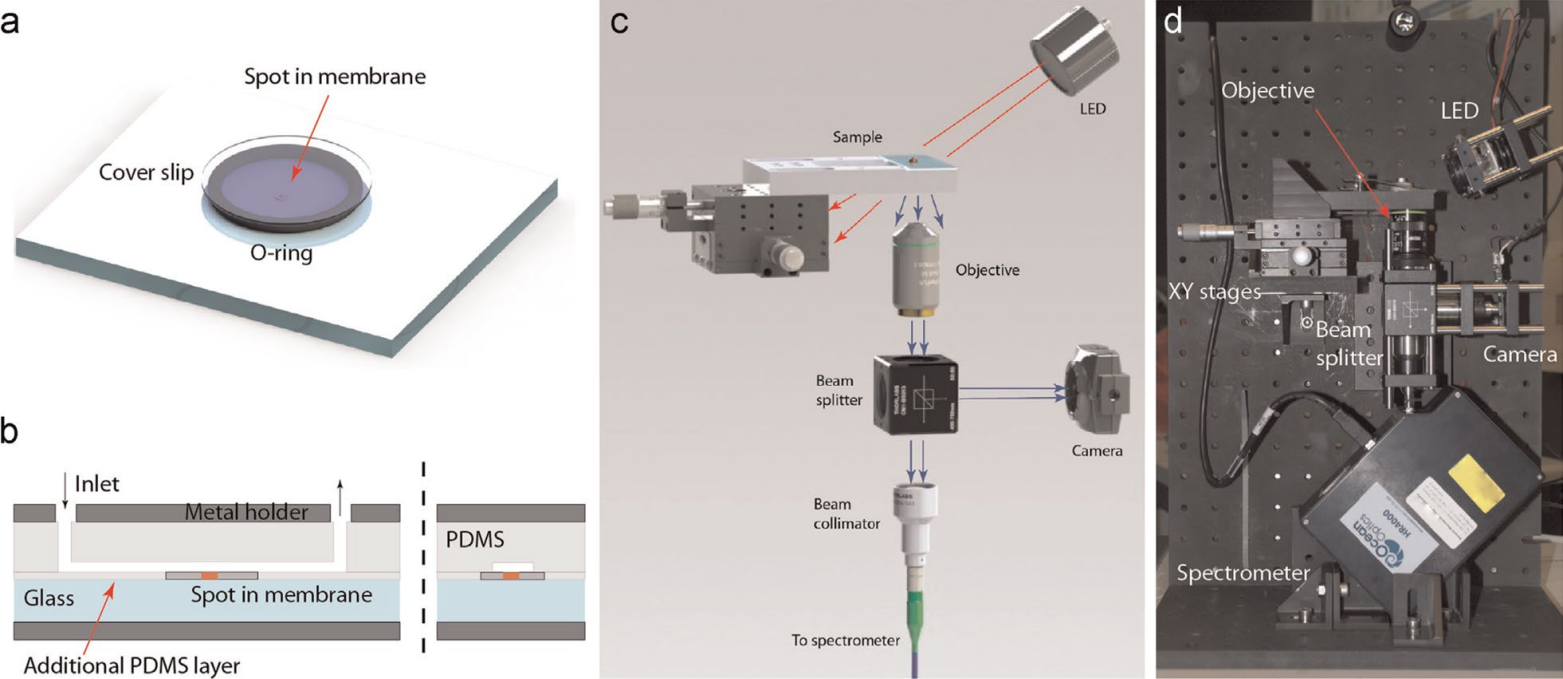
Portable setup (**a**) closed chamber for static measurements, **b** microfluidic channel for flow experiments, **c** schematic drawing of the portable setup and **d** front view photograph. Reprinted with permission from (*Biosensing and Bioelectronics* 2015; 68: 245–252). Copyright (2015) Elsevier

**Fig. 9:**
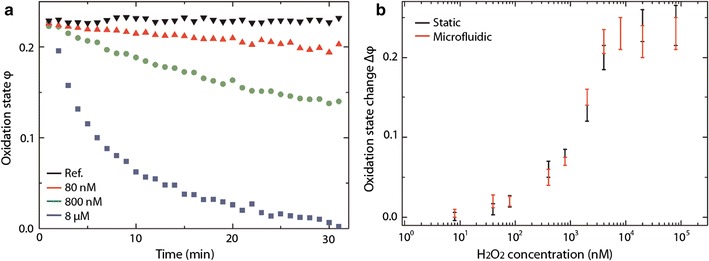
**a** Time evolution of the oxidation coefficient φ for different H_2_O_2_ concentrations in the static regime. **b** Differential oxidation coefficient Δφ vs. H_2_O_2_ concentration in the static and microfluidic regime. Reprinted with permission from (*Biosensing and Bioelectronics* 2015; 68: 245–252). Copyright (2015) Elsevier

**Fig. 10:**
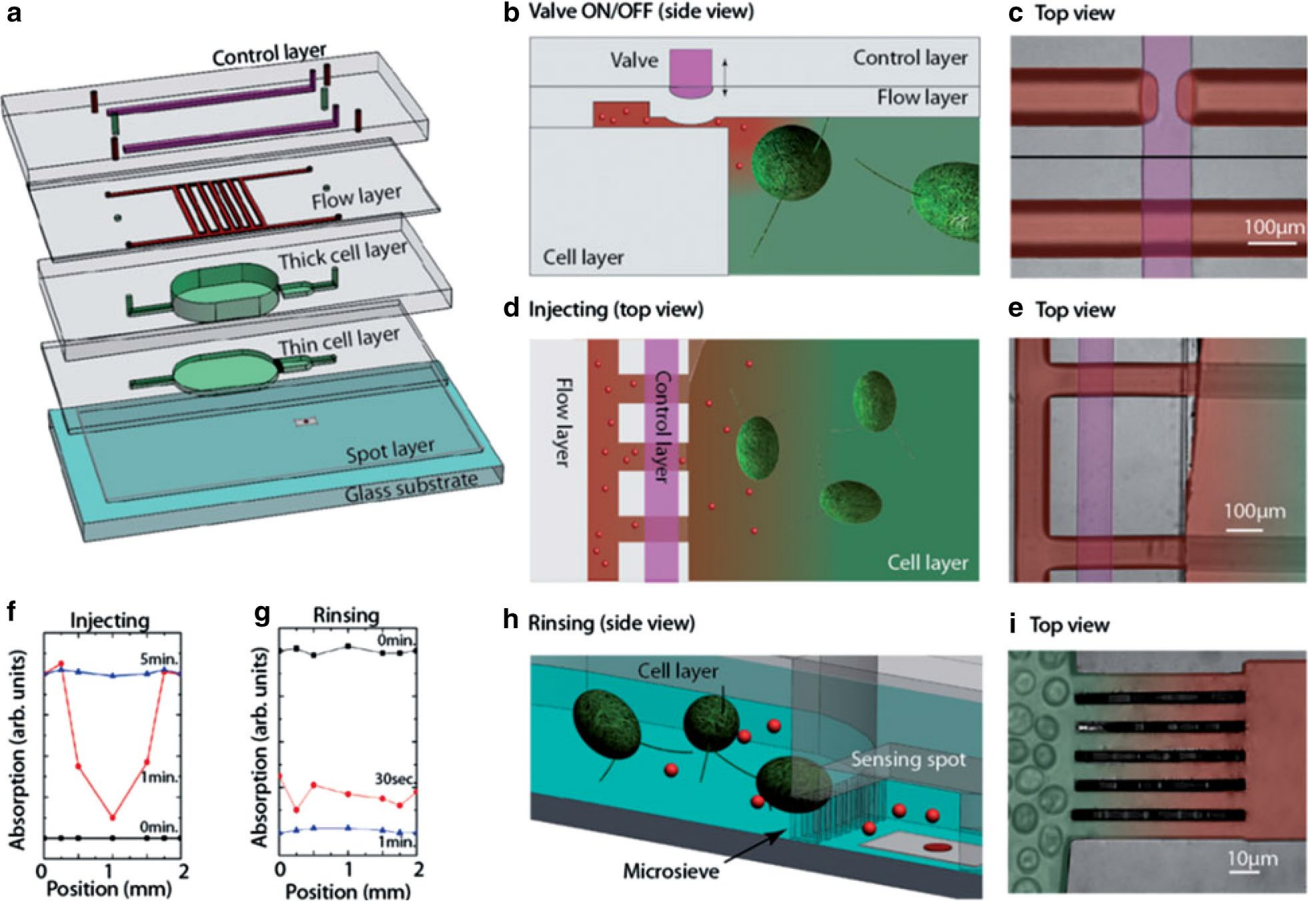
**a** Conceptual design of the multilayered microfluidic configuration, **b**–**e** principle of operation of the microfluidic valves, schematic drawings and photographs, **f**, **g** time evolution of the analyte concentration for filling and rinsing action, **h**, **i** schematic drawing and photograph of the micro sieve. Reprinted with permission from (*Nanotoxicology* 2016; 10: 1041–1050). Copyright (2016) Taylor & Francis

To step towards reliable and sensitive routine H_2_O_2_ measurements, a portable setup containing a multiscattering sensing element was built (Fig. 8) [82]. An aqueous solution of cyt *c* was spotted onto a porous filter membrane using a microarray robot with a delivery volume of 5 nl of 4 mM cyt *c* solution. Subsequently, the cyt *c* was crosslinked with vaporous glutaraldehyde in order to retain the cyt *c* in the membrane. Using the membrane approach the reproducibility of the amplification was remarkably improved compared to the aggregates described in the previous section. A closed chamber delimited by an o-ring and two glass cover slips was employed to carry out static experiments (Fig. 8a). The sensing element was placed at the bottom of the chamber prior to the measurements. Figure 9a shows the time evolution of φ in the static regime for different H_2_O_2_ concentrations in PBS buffer solution [82]. Measurements performed in this configuration exhibit a signal enhancement due to multiscattering, on the order of 5. In a further step the configuration was extended with a multi-layered microfluidic arrangement containing micro-valves and sieves [83], enabling more complex experimental sequences; for instance exposure/rinsing steps to study recovery or sensitisation of bioorganisms. Schematic overview and photographs of the principle of the portable oxidative stress sensor (POSS) are displayed in Fig. 10. The implementation of microsieves offers the possibility to perform experiments with non-adhering bioorganisms such as algae, which are retained in the reaction chamber as illustrated in Fig. 10h, i. The sensing element is placed in the microfluidic channel in order to minimise possible interferences between organisms and analytes. Figure 9b shows the differential oxidation state coefficient Δφ vs. H_2_O_2_ concentration for the static and microfluidic regime. Δφ defined as the difference between the initial value of φ_t = 0_ and the value at time t: $\Delta \varphi = \varphi_{t = 0} \, - \,\varphi_{t}$. The calibration curve resembles a sigmoidal shape when increasing H_2_O_2_ concentration, which is typical for ligand binding assays and can be fitted using a 4-parameter logistic model [84]. For the given configuration with a porous membrane a LOD of 40 nM of H_2_O_2_ was achieved [82]. Exposing the sensing element to reducing agents the cyt *c* alters from its ferric Fe^III^ state to its ferrous Fe^II^ state. Hence, after reducing an oxidised sensing element can be reused. This has been shown by exposing the sensing spot to AA. Four consecutive oxidation/reduction cycles were carried out without lowering the performance of the sensor [82]. Furthermore, glucose and H_2_O_2_ and lactate and H_2_O_2_ were simultaneously measured adding glucose (GOx) and lactate oxidase (LOx), respectively, for the enzymatic conversion into H_2_O_2_ [Eq. (4)] [40]. Thus, to avoid that the fast conversion already takes place in the solution the oxidase was incorporated inside the sensing element. In practise, a mixture of oxidase and cyt *c* was deposited onto the filter membrane prior to crosslinking with glutaraldehyde, as described above for cyt *c*. An unambiguous measurement of glucose and lactate concentrations requires simultaneous measurements of the substrate (glucose and lactate in the present cases) and H_2_O_2_ with subsequent subtraction of the background H_2_O_2_ contribution. For the sake of completeness, it should be mentioned that, due to diffusion issues, interferences were observed when placing the sensing elements for the substrate and H_2_O_2_ in the same chamber. This problem was solved by adapting the microfluidic configuration to separate the sensing elements [40]. Finally, LODs as low as 240 and 110 nM for lactate and glucose, respectively, were achieved for the configuration at hand.

Here ENM-induced H_2_O_2_ excretion by cells exposed to ENMs was monitored with a recently developed optical biosensor in a portable setup (POSS; portable oxidative stress sensor) specifically designed for field experimentation [82]. In this way, POSS may contribute to the elucidation of ENM-specific pro-oxidant interactions with cells and thus help to narrow the gap between material innovation and sound risk assessment.

## Selected applications to probe the pro-oxidant effect of nanoparticles to microalga *C. reinhardtii*

To demonstrate the performances of the developed sensing tool, the pro-oxidant effects of CuO and TiO_2_ nanoparticles to green alga *C. reinhardtii*, a representative model AMO are presented [32, 85] together with measurements of the potential to generate abiotic ROS as well as oxidative stress and membrane damage. These two ENMs were chosen since they have different properties—CuO nanoparticles have a tendency to dissolve, while nano-TiO_2_ is rather inert; (ii) both have photocatalytic properties; (iii) nano-CuO is with relatively high toxic potential [86], while nano-TiO_2_ is moderately toxic; (iv) they are of high environmental relevance given their increasing use in different products.

The nanoparticle-induced cellular pro-oxidant process in *C. reinhardtii* were studied using the newly developed cytochrome *c* biosensor for the continuous quantification of extracellular H_2_O_2_ and fluorescent probes (CellRoxGreen for oxidative stress and propidium iodide for membrane integrity [32, 41, 87]) in combination with flow cytometry. Both the dynamics of abiotic (ENM only) and biotic (ENM + cells) pro-oxidant processes related to the exposure of *C. reinhardtii* to nano-CuO and nano-TiO_2_ are present below.

### Nano-CuO

*Chlamydomonas reinhardtii* were exposed to CuO nanoparticles in five different media, namely TAP, MOPS, OECD, MES and Geneva lake water [85] and the biological responses including growth, size increase, chlorophyll autofluorescence, intracellular ROS and membrane damage were quantified.

**Fig. 11:**
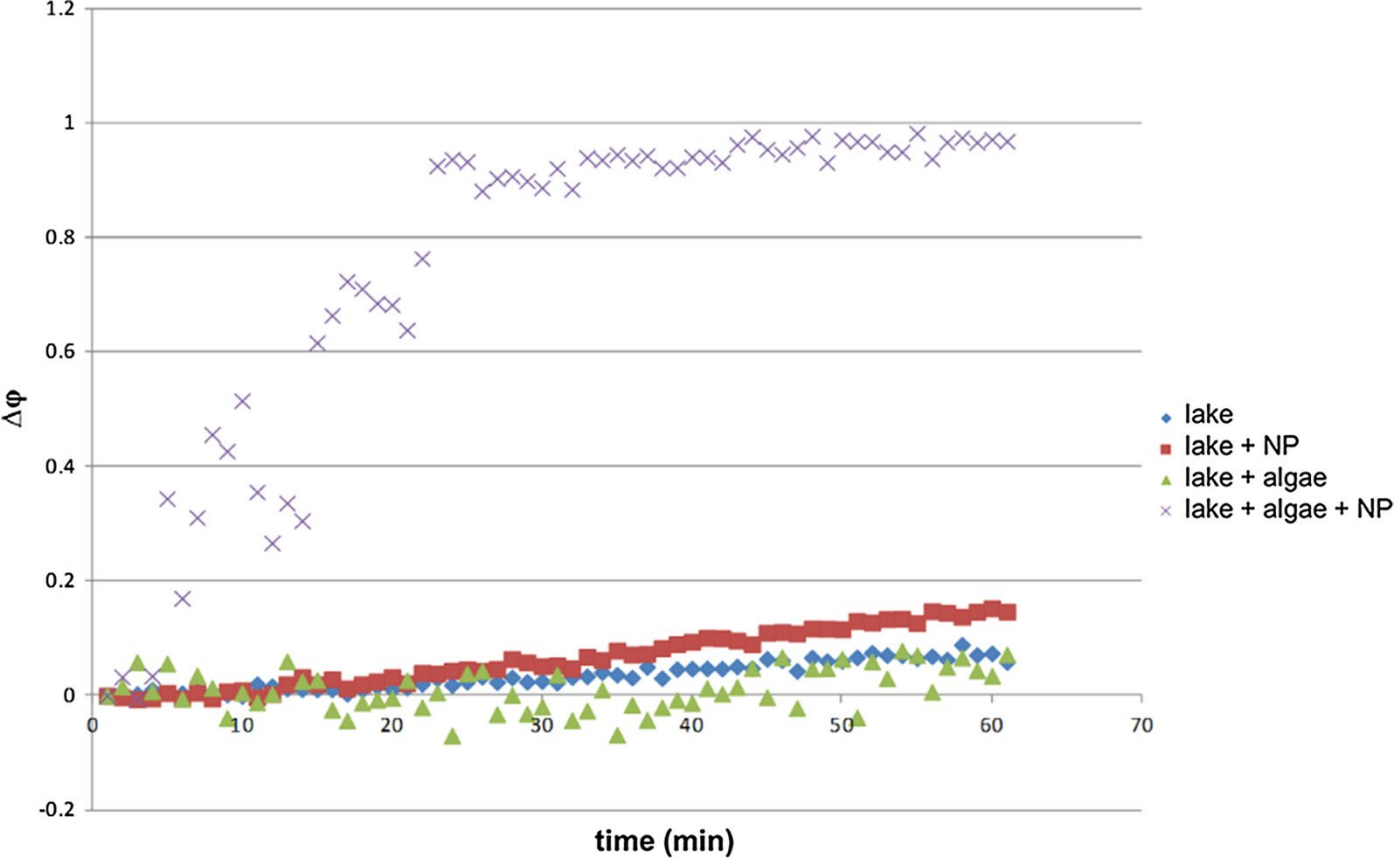
Time evolution of the differential oxidation coefficient Δφ during an exposure for 60 min to nano-CuO, *C. reinhardtii* and nano-CuO and *C. reinhardtii* in lake water. A control experiment was carried out in lake water

The concentration of Cu ions dissolved from the nano-CuO in the different media increased in the order: MOPS < MES < Geneva lake water < OECD < TAP. Nano-CuO exposure induced oxidative stress and membrane damage, but the intensity of the effects was susceptible to medium and exposure duration [40]. Comparison of the exposure of *C. reinhardtii* to nano-CuO and released Cu^2+^ revealed that in all but one of the five different exposure media free ionic copper was likely the main toxicity-mediating factor. However, a threshold concentration of Cu^2+^ must be reached for biological effects to occur. However, a nano-CuO particle effect was observed in cells exposed in the Good’s buffer MOPS, in which nano-CuO dissolution was very low. These findings highlight how the dominant toxicity mediating factors change with exposure medium, time and the biological endpoint considered and thus demonstrate that nanotoxicity is a highly dynamic process. Furthermore, the observed ROS generation and oxidative stress observed in *C. reinhardtii* exposed to nano-CuO in lake water, were in line with the increasing extracellular H_2_O_2_ determined using the POSS (Fig. 11). Abiotic H_2_O_2_ formation by nano-CuO was also observed, but the values were much lower than those found in the presence of algae. Simultaneous exposure of *C. reinhardtii* to nano-CuO and simulated solar light induced synergistic effect in ROS generation, whereas exposure to ionic copper and the same solar simulated light conditions resulted in antagonistic effects [41, 87]. No measurable alterations in nano-CuO aggregation, copper dissolution or abiotic ROS production were found under the tested light irradiations suggesting that the synergistic effects are not associated with light-induced changes in nano-CuO properties in the exposure medium [40, 41]. Nano-CuO toxicity to microalgae is generally recognized to be associated with the amount of copper released by the nanoparticles [41]. However, the combined effects observed for light irradiation and CuO-NPs could not be explained with the measured copper dissolution suggesting that under stressful light conditions other mechanisms of actions might be involved.

### Nano-TiO_2_

The nano-TiO_2_ exposure experiments were performed in MOPS and water sampled from lake of Geneva [32]. The observed pro-oxidant effects were strongly dependent on the exposure concentration and medium. In lake water exposures the proportion of cells affected by oxidative stress increased with the concentration of nano-TiO_2_, with highest responses obtained for algae exposed to 100 and 200 mg L^−1^ nano-TiO_2_. Similarly, membrane damage predominantly occurred in lake water rather than in MOPS. UV light pre-treatment of TiO_2_ enhanced median intracellular ROS levels in lake water exposure while no significant effect was found in MOPS.

**Fig. 12:**
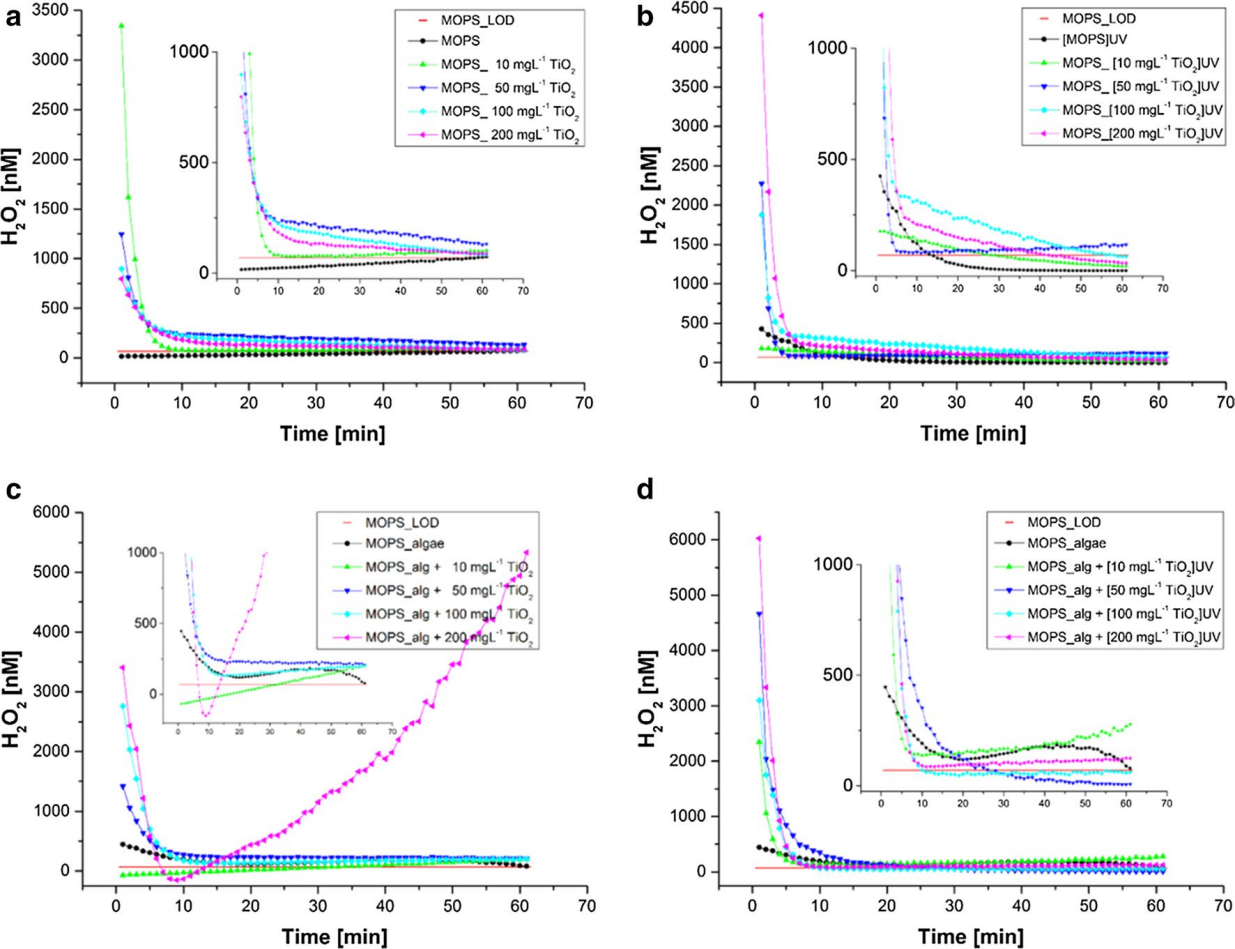
Extracellular H_2_O_2_ [nM] (*c*_*H2O2*_) produced during 60 min by four nano-TiO_2_ concentrations with (**b**, **d**) and without UV pre-treatment (**a**, **c**) in abiotic (**a**, **b**) and biotic (**c**, **d**) conditions in the MOPS buffer: nano-TiO_2_ only (**a**), nano-TiO_2_ after 20 min UV pre-treatment (**b**), algae exposed to nano-TiO_2_ (**c**) and algae exposed to UV pre-treated nano-TiO_2_ (**d**). The *horizontal red line* represents the LOD and *insets* depict enlargements of the respective 0–1000 nM concentration range Reprinted with permission from (*RSC Advances*  2016; 6: 115271–115283). Copyright (2016) Royal Society of Chemistry

In MOPS H_2_O_2_ concentrations (*c*_*H2O2*_) determined using POSS were highest at the start and decayed to values close to the LOD after 60 min exposure (Fig. 12) in all treatments. *c*_*H2O2*_ values were higher in UV pre-treated samples at nearly all concentrations (except 10 mg L^−1^ nano-TiO_2_). The initial *c*_*H2O2*_ peaks are possibly due to the formation of hole/electron pairs and their subsequent photocatalytic reaction with H_2_O and O_2_ at the surface of the nano-TiO_2_ particles [88]. Results suggest that nano-TiO_2_ behaves as both peroxide source and sink through photocatalytic reactions at the surface of the nanoparticles. Experiments carried out with lake water did not exhibit initial peroxide peak concentrations after sonication. This may be explained by ROS quenching species in the form of dissolved organic matter (DOM), which, in contrast to MOPS, are present in lake water.

The biotic exposure experiments revealed higher decay rates of the initial peaks at the beginning of the experiments, suggesting a peroxide annihilation by algae.

Overall, our findings showed that (i) irrespective of the medium, agglomerated nano-TiO_2_ in the micrometer size range produced measurable abiotic H_2_O_2_ concentrations in biologically relevant media, which is enhanced by UV irradiation, (ii) *c*_*H2O2*_ undergo decay and are highest in the first 10–20 min of exposure and (iii) the generation of H_2_O_2_ and/or the measured H_2_O_2_ concentration is a dynamic process modified by the ambient medium as well as nano-TiO_2_ concentrations and the presence of cells.

Comparison of the extracellular H_2_O_2_ measurements and intracellular oxidative stress [32, 82] further showed significant differences between extracellular and intracellular pro-oxidant processes. Indeed, an increase of the intracellular oxidative stress was found under the conditions where no significant increase in extracellular biotic H_2_O_2_ was measured. The above observation indicates that extracellular H_2_O_2_ measurements cannot directly serve as a predictor of cellular pro-oxidant processes or oxidative stress in *C. reinhardtii*, however, they provide valuable information about the extracellular dynamics of the most stable ROS in the extracellular medium.

## Extracellular H_2_O_2_ measurements during altering illumination regimes

It is well known that light conditions influence the metabolic activity of algae and therefore cellular ROS generation [89, 90]. ROS released by photosynthetic organisms generally originate from the photosystems II and I [89, 90] (PSII and PSI) located in the thylakoid membrane of the chloroplast. Disturbances of the electron transport chain from PSII to PSI favour reduction of molecular oxygen O_2_ to O_2_^−^ which triggers a reaction cascade leading to the formation of OH and H_2_O_2_ [91]. According to previous studies, chloroplast derived H_2_O_2_ is able to diffuse out of the chloroplast [92] and through the cell walls and is, therefore, present in the extracellular media. Here, we examined the dynamics of extracellular H_2_O_2_ during altering illumination regimes. *C. reinhardtii* in model medium were exposed to 100 nM of Cd^2+^ in different light conditions [18].

5 $$C. reinhardtii + {\text{Cd}}^{2 + } \quad{\underrightarrow {light}}\quad{\text{extracellular H}}_{2} {\text{O}}_{2}$$

**Fig. 13:**
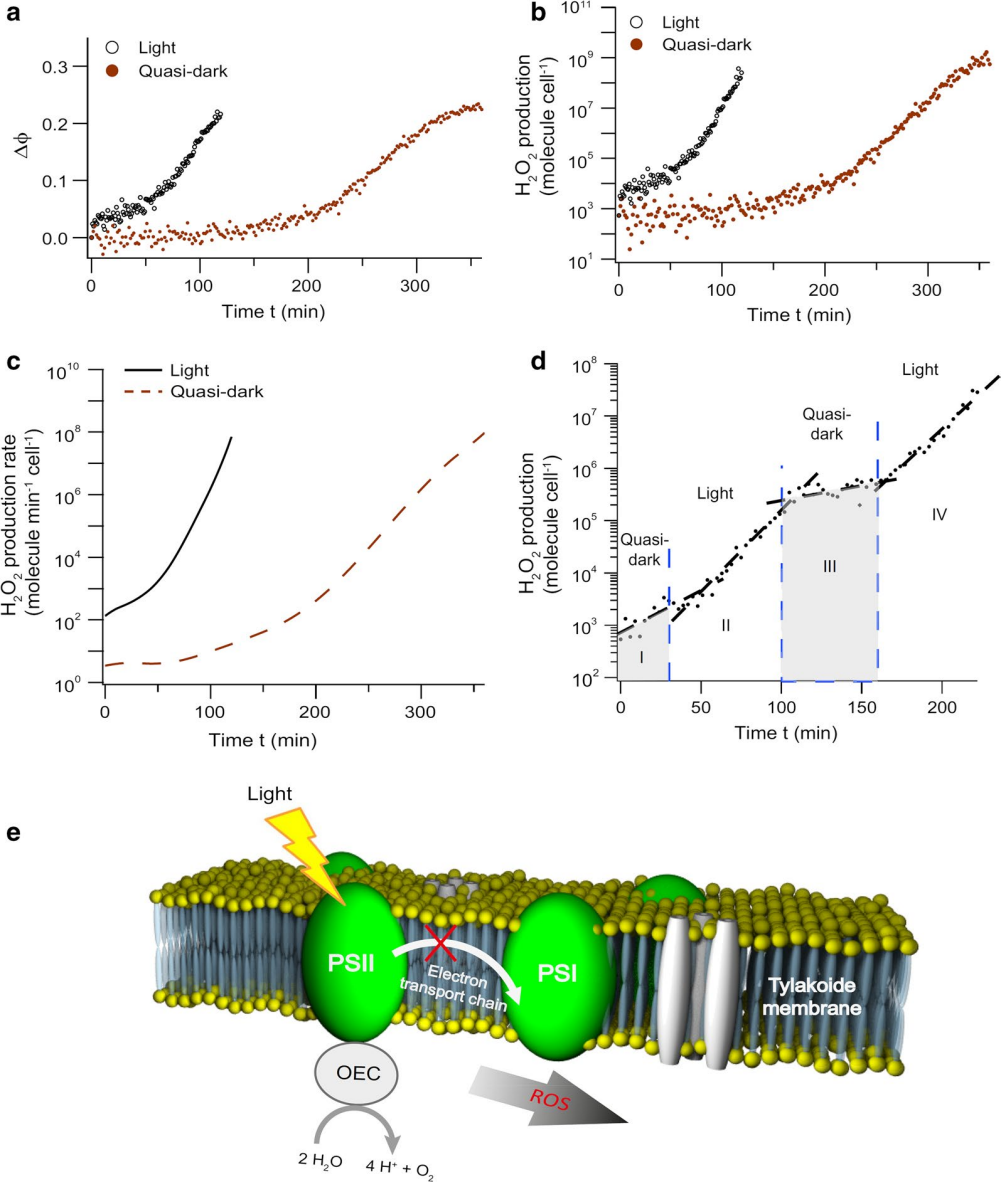
Dark- and light-adapted *C. reinhardtii* are exposed to 100 nM of Cd(II). Time evolution of (**a**) the differential oxidation state coefficient Δϕ, **b** the H_2_O_2_ production and **c** the H_2_O_2_ production rate. After injection of Cd(II) the light-adapted algae under illumination start excreting H_2_O_2_ without delay, whereas there is a production delay under dark conditions. **d** ROS production for dark-adapted algae exposed to 100 nM of Cd(II) when the illumination is successively turned on and off during the measurement. **e** These data support the following action mechanism of Cd(II) on the photosynthetic apparatus of *C. reinhardtii*: Cd(II) binding to the plastoquinone pool disturbs the electron transport chain between PSII and PSI. Upstream, the light driven electron extraction from oxygen evolving complex (OEC) remains functional and generates light-dependant ROS at the PSII acceptor side. Reprinted with permission from (*Scientific Reports* 2013; 3: 3447). Copyright (2013) Nature Publishing Group

Figure 13 indicates an enhanced H_2_O_2_ production rate and no production delay under light conditions suggesting a correlation between ROS regulation and the activity of the photosystems.

## Recovery and sensitisation

In contrast to end-point measurements, sensitive and non-invasive continuous H_2_O_2_ measurements enable the investigation of recovery and sensitisation. To demonstrate the practicability of such experiments the *C. reinhardtii* were repeatedly exposed to Cd^2+^, using a microfluidic configuration as described above [83]. Cd^2+^ concentrations are typically <10 nM in fresh water. However, higher concentrations of Cd^2+^ were found in the exposure media containing CdSe quantum dots [5] or CdTe/CdS [34].

**Fig. 14:**
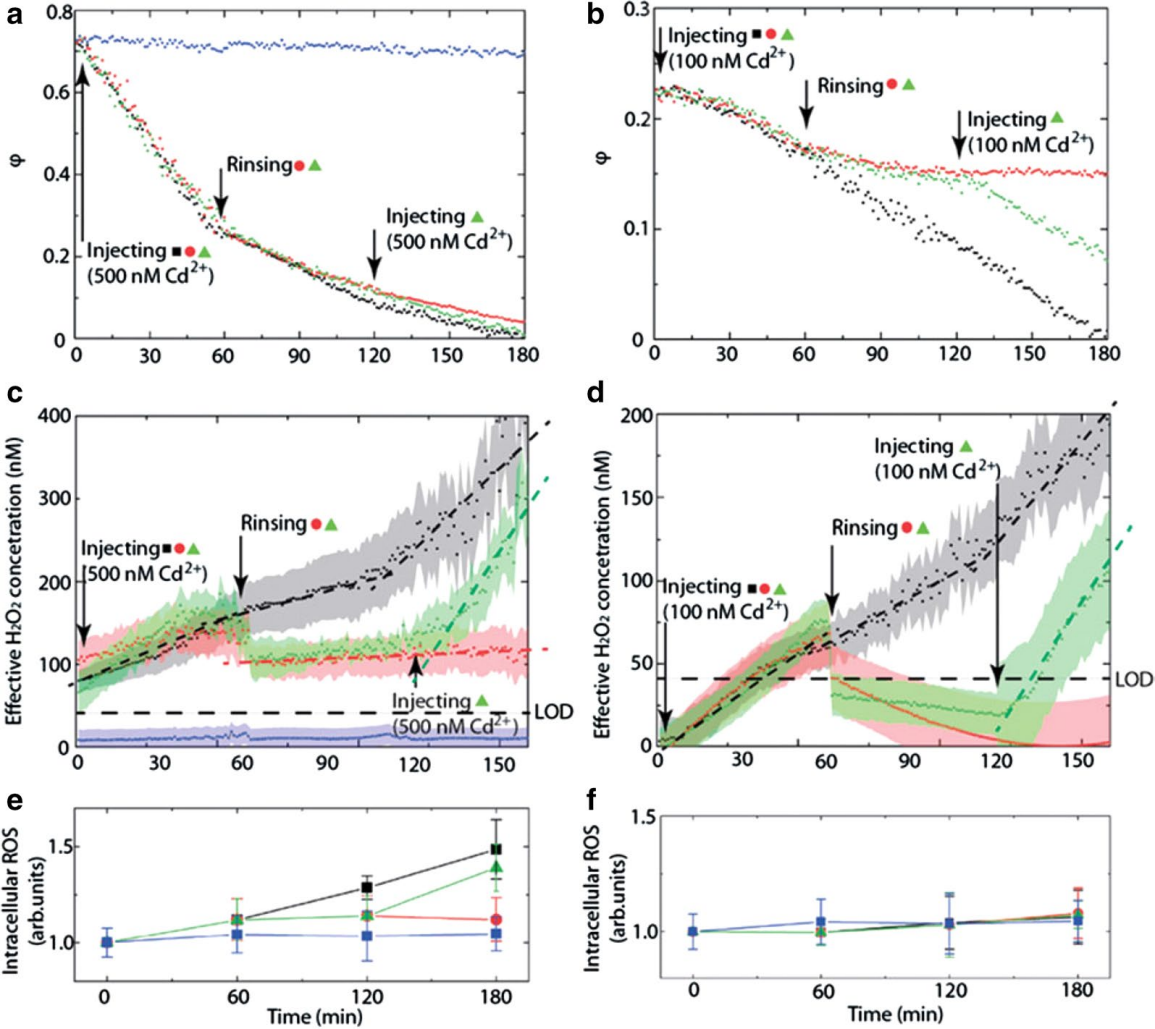
Algae exposure to Cd^2+^. Oxidative state coefficient *φ* versus time for: **a** 500 nM and **b** 100 nM exposure cycles. **c**, **d** Extracellular H_2_O_2_ concentration *C*_*H2O2*_. Intracellular ROS measured a fluorescence method for **e** 500 and **f** 100 nM Cd^2+^ exposures for identical cycles as in **a** and **b**. Reprinted with permission from (*Nanotoxicology* 2016; 10: 1041–1050). Copyright (2016) Taylor & Francis

Extracellular H_2_O_2_ concentrations were measured while *C. reinhardtii* were exposed to 100 and 500 nM of Cd^2+^ [step (1)]. A subsequent rinsing [step (2)] and further exposure to Cd^2+^ [step (3)], even at 100 nM, exhibits an increased H_2_O_2_ production rate compared to the previous exposure (Fig. 14).

1. 1st exposure of *C. reinhardtii* to Cd^2+^ → H_2_O_2_ production
2. Rinsing
3. 2nd exposure of *C. reinhardtii* to Cd^2+^ → increased production rate of H_2_O_2_

This shows that exposure to even low concentration of Cd^2+^ leads to a sensitisation of exposed cells, thus suggesting an adverse impact on the health of microorganisms. In parallel, intracellular ROS was assessed based on the fluorescence intensity of de-esterified H_2_DFC-DA [93]. At high Cd^2+^ concentrations (500 nM) intra- and extracellular measurements correlated very well, confirming the suitability of extracellular H_2_O_2_ measurements as indicator of cellular stress. However, unlike extracellular H_2_O_2_ concentrations, intracellular levels remain stable in the 100 nM exposure, suggesting an efficient ROS/AOX regulation through the cell walls.

## Conclusions and outlook

This review paper provides a short overview on nanoparticle toxicity for aquatic microorganisms based on the paradigm of oxidative stress and highlights the recent developments of an optical biosensor based on absorption measurements of cyt *c* for the sensitive, non-invasive and continuous measurement of H_2_O_2_. The use of this new tool for studying the pro-oxidant effects of ENMs to aquatic microorganisms was demonstrated by exposing the representative aquatic microorganism *C. reinhardtii* to nano-CuO and nano-TiO_2_ in various exposure media and under different light treatments. Sensitive continuous measurements of extracellular H_2_O_2_ provided valuable information on both the potency of the studied nano-CuO and nano-TiO_2_ to generate ROS as well as on the mechanisms of toxicity. The results were in good agreement with the oxidative stress and membrane damage results obtained under the same conditions using a combination of fluorescent staining with flow cytometry. The developed biosensor allows rapid measurement of the rate and amount of H_2_O_2_ measured in the extracellular medium in response to cell exposure to ENMs. Hence, detailed knowledge of the dynamics of H_2_O_2_ excretion can provide valuable insights into complex biological responses. The development of the portable setup and the multi-layered microfluidic chip with an integrated optical sensor for the continuous sensitive detection of extracellular H_2_O_2_ opens novel avenues for new types of exposure experiments, leading to a better understanding of ROS biology as well as to numerous opportunities for nanoecotoxicological studies. Developing and employing new sensing tools and methods enables conducting experiments under more realistic conditions such as environmental relevant concentrations, aged nanomaterials and simultaneous exposure to various stressors. Furthermore, studying the dynamics of cellular metabolites leads to new insights in the extremely complex adverse outcome pathways.

## Abbreviations

ENMs: engineered nanomaterials
ROS: reactive oxygen species
AOX: antioxidant
AMO: aquatic microorganism
MEAS: multiscattering enhanced absorption spectroscopy
OPL: optical path length
LOD: limit of detection
POSS: portable oxidative stress sensor
AuNp: gold nanoparticle
cyt *c*: cytochrome *c*
PSI: photosystem I
PSII: photosystem II
MES: 2-(*N*-morpholino)ethanesulfonic acid
MOPS: 3-(*N*-morpholino)propanesulfonic acid
TAP: N-Tris[hydroxymethyl]methyl-3-aminopropanesulfonic acid
OECD: OECD standard media
DOM: dissolved organic matter
SOD: superoxide dismutase
CAT: catalase
GR: glutathion reductase
AA: ascorbic acid
HA: humic acid
SRFA: Suwannee River fulvic acid
EDTA: ethylenediaminetetraacetic acid
